# Marriage in displacement: Gendered (self)resettlement strategies of Syrian women in Egypt

**DOI:** 10.1111/1468-4446.13145

**Published:** 2024-09-04

**Authors:** Dina M. Taha

**Affiliations:** ^1^ Department of Sociology and Anthropology Doha Institute for Graduate Studies Doha Qatar; ^2^ Centre for Refugee Studies York University Toronto Ontario Canada

**Keywords:** agency, Egypt, forced migration, gender, marriage, resettlement, Syria

## Abstract

Drawing on fieldwork data among Syrian refugee women marrying Egyptian men amid forced migration, I explore how displacement reshapes the meaning and purpose of marriage. Many such unions, often customary or polygamous, provoke comparisons to forced marriage and gender‐based violence. Bypassing the reductive exploitation and static narratives, I ask: How does displacement alter refugee women's perceptions of marriage's purpose? And can marriage serve as a strategic tool for (self)resettlement? This investigation urges us to reevaluate the existing range of resettlement options and criteria, offering fresh perspectives on marital strategies post‐displacement. Rather, similar marriages often stem from both affective and practical considerations, challenging colonial dichotomies (e.g., agent/victim) and reinstating the role of factors such as social capital in the trajectories of the uprooted. This study expands understanding of gendered and Othered refugee experiences, highlighting marriage's transformative role in forced displacement and resettlement. It contributes to ongoing discussions on marriage, displacement, and resettlement, urging a nuanced approach that acknowledges the complexities of refugee agency and adaptation.

## 1 INTRODUCTION

Marriage between Egyptian men and Syrian refugee women has drawn media attention, frequently been labeled as exploitative. Many such marriages, often unregistered or polygamous, have been criticized as gender‐based violence (GBV) by feminist advocacy groups and media outlets (Barkan, 2012; Bayoumi, 2013; Fajry, 2012; Hassan, 2015; Heinrich Boll, 2013). Although the full scale of this issue remains uncertain and untracked, numerous human rights organizations, nonprofits, and media outlets have reported and criticized these arrangements (See Bayoumi, 2013; Fajry, 2012; Heinrich Boll, 2013; Hassan, 2015; Barkan, 2012). For instance, despite the absence of the original data, secondary sources indicate that the National Council for Women's Protection in Egypt reported about 12,000 Syrian women engaging in such marriages in 2013 (Natour, 2016; Egypt Independent, 2013; UNHCR Data Portal, 2013). Renowned publications including The Egyptian weekly Al‐Ahram Al‐Arabi, The Algerian daily Al‐Fajr, and The London‐based Saudi Al‐Hayat have portrayed these Syrian refugee women as “easy prey and a valuable catch,” commending those brave enough to confront the exploitation of refugee women (Barkan, 2012).

Gender in the Middle East, especially in the Arab world, has been Orientalized and even weaponized for decades (Abu‐Lughod, 2002; Razack, 2004). Critical feminist perspectives reveal how colonial and Orientalist narratives have fixated on sexuality to Other and validate a white savior complex and a colonial civilizing mission (Ahmed, 1992; Farahani & Thapar‐Björkert, 2020). Drawing on such critical perspectives, specifically by employing a decolonial intersectional reading, this paper explores the complex realities of Syrian refugee women marrying Egyptian nationals post‐displacement, challenging dominant narratives of exploitation and offering a nuanced understanding of marriage and family in the Arab context. It aims to illuminate the region's multifaceted nature of gendered subjectivities and non‐hegemonic societal structures.

As such, this paper aligns with literature challenging Eurocentrism and colonial legacies in knowledge production. Decolonization aims to offer alternative worldviews that respect diverse experiences and subjectivities. Building on foundational work such as Said's “Orientalism” (1978) and Fanon's (2002) critique of colonial systems and call for a radical reimagining of society, decolonial frameworks recognize the inequality in knowledge production and cultural representation, advocating for a more inclusive worldview that respects diverse experiences and subjectivities (Mohanty, 1988; Spivak, 1988; Tiostanova & Mignolo, 2012). By recognizing and linking such work to refugee research, I seek to explore Other(ed) ways of being while emphasizing the inadequacy of some hegemonic and normative notions, such as the nuclear family, in fully explaining refugee experiences. To this point, Sherene Razack (2004) argued that it is ubiquitous when adopting a “Western feminist worldview,” with its cultural and historical specificity, to fall into “cultural deficit explanations” when attempting to understand and explain non‐Western women's experiences, describing them often as “overly patriarchal and inherently uncivilized” (p. 129).

This paper aims to foreground Syrian refugee women's narratives to acknowledge marginalized and Othered experiences, ways of knowing, and ways of existing in the world. The analysis challenges Western‐centric worldviews and what has been conventionally and hegemonically constituted as morally correct. It subsequently centers gendered, self‐initiated, and innovative resettlement options that pose critical questions to refugee studies and gender studies by shifting the discourse toward self‐authorized modes of protection (Hyndman & Reynolds, 2020) and “self‐rescue” resettlement options (Kyriakides et al., 2019).

Based on fieldwork in Egypt in 2017, I explore the role of marriage in Syrian refugee women's (self)resettlement strategies in the face of a restricting refugee regime. While this case study focuses on Egypt, a country without an encampment system, similar practices occur in other countries like Turkey, Lebanon, and Saudi Arabia (Bayoumi, 2013; Fajry, 2012). These marriages are facilitated through marriage brokers, social media, and religious groups (Barkan, 2012; Geha, 2013; Hassan, 2015). Instead of framing them as GBV or mere exploitation, this study examines how displacement changes perceptions of marriage by which it can serve as a strategic approach to (self)resettlement. I ask, how might displacement change refugee women's perceptions of the meaning and purpose of marriage? How can marriage, in such cases, serve as a strategic and agentive choice for resettlement? This examination prompts us to reconsider existing resettlement options and parameters, emphasizing the transformative potential of marriage in forced displacement contexts, particularly in gaining lost social capital. Such marriages often stem from a mix of affective and pragmatic interests, challenging the notion of agency as solely driven by self‐interest and accentuating the role of the intimate and the private in shaping migration and displacement experiences.

I begin with a discussion of the research methods, followed by a brief overview of the circumstances of Syrian refugee women in Egypt, which has prompted some to consider marriage as a means of survival. I commence the analysis by anchoring my argument in previous research exploring the impact of displacement on perceptions of marriage and gender norms. Subsequently, I delve into three aspects that illustrate how marriage has been redefined or repurposed after displacement, making it a practical choice for self‐resettlement. The first aspect focuses on *remarriage*, which has become a viable option post‐displacement, often unattainable and undesirable in Syria for various reasons. The second and third aspects trace the transformation of the ideal marriage image, encompassing unconventional forms of marriage for many respondents, namely: *‘urfi* (customary or unregistered) marriages and *polygamy*. By spotlighting Othered/Orientalized marriage practices and ways of life, this discussion illuminates diverse experiences of gendered displacement. Additionally, it encourages a deeper understanding of non‐Western gendered relations and their “eligibility to exist” beyond hegemonic discourse (Kyriakides et al., 2019).

## 2 METHODOLOGY

Drawing upon doctoral fieldwork in the summer of 2017, I conducted semi‐structured interviews over four months in Cairo and Alexandria, areas hosting the largest number of Syrian refugees residing in Egypt. Employing personal connections, snowball sampling, and social media, I Interviewed 30 Syrian women, nine Egyptian husbands, and seven mothers‐in‐law. I sought to represent all socio‐economic levels as well as age groups and marital statuses. Even though it was primarily a convenience sample, in the middle of the fieldwork, I made some adjustments by incorporating elements of purposive sampling by seeking women from the upper‐middle and upper classes who had initially been underrepresented. In doing so, I sought to balance the initial recruitments that mainly were from lower‐middle and lower classes by seeking references through social media and acquaintances.

The author's positionality played a role in shaping the data collection and analysis. My Egyptian upbringing and Muslim background provided cultural insight, enhancing the depth of the study. Being an Arab mother has facilitated building trust and rapport among the women respondents compared to the men, many of whom were also mothers, and fostered a more in‐depth conversation and more meaningful prompts and reflections beyond the initial scripted questions. Nevertheless, being a non‐refugee, though a migrant to a Western country, my urban upper‐middle‐class socio‐economic status and Western education contrasted with my participants, potentially affecting rapport and understanding, creating different points of tension but also empathy and common ground. This dynamic required constant reflexivity on my part, ensuring that the voices and interpretations of the women were communicated authentically and respectfully.

To ensure the voices of the respondents were authentically represented, I regularly relied on extensive and long direct quotations. This approach aimed to closely connect the research with their real‐life experiences while reducing biased interpretations and highlighting the collaborative nature of data creation between the researcher and participants. Handling the translation from Arabic to English was a critical task. It involved maintaining cultural relevance and context and recognizing that translations inherently capture only part of the original meaning. To mitigate this, the study provided contextual details and retained certain Arabic expressions to preserve specific nuances lost in English. The analysis employed narrative and discourse analysis methods to reveal both overt and covert signals. It delved into life trajectories, personal agency, and how power structures and dominant discourses shape individual experiences and perceptions. The discourse analysis particularly focused on understanding how societal narratives impact and govern the lived experiences of Syrian refugee women and Egyptian men.

## 3 SYRIAN REFUGEE WOMEN IN EGYPT

Egypt is home to over 5 million refugees and migrants (Karasapan, 2016), including 352,000 registered refugees and asylum seekers where Syrians remain the largest group (UNHCR Egypt Factsheet, 2023). This number is a huge underestimation since most Syrians arrive in Egypt on Tourist visas that they can easily convert to work permits or simply keep renewing (Ayoub & Khallaf, 2014). Egypt does not have a policy of encampment and, historically, Cairo has hosted many foreigners and refugees, including Armenians, Palestinians, and Sudanese (Ayoub & Khallaf, 2014). In this respect, at least formally, refugees have the right to freedom of movement and integration (Ayoub & Khallaf, 2014), especially if they are from Arab ethnic and linguistic backgrounds such as Palestinians and Iraqis. However, changes in public and political attitudes have shaped the reception and perceptions of Syrian refugees and their arrival in Egypt since 2012.

Furthermore, most refugees face numerous challenges in Egypt, including limited social and economic opportunities and the high cost of living in an economically challenged and politically polarized environment (ILO, 2018). A UNHCR report revealed that the majority of Syrian refugees in Egypt live in relative deprivation, with 93% unable to afford basic daily expenses, and Syrian households headed by women are particularly vulnerable due to cultural barriers and protection concerns, leading to lower employment rates (UNHCR Egypt, 2016). Although Egypt is a signatory to the 1951 UN Convention Relating to the Status of Refugees, it withdrew some fundamental rights for refugees, such as the right to work (Hetaba et al., 2020). Consequently, most Syrians work in the informal sector, facing exploitation and housing market challenges (Bidinger et al., 2014). While Syrian children have equal access to Egyptian public schools, discrimination and the quality of education sometimes lead families to opt for community‐organized or private schools (PRM report, 2012). The Syrian refugee community in Egypt has made entrepreneurial efforts, especially in the food industry, contributing to the local economy (Ayoub & Khallaf, 2014).

On the issue of marriage, Nahla, a journalist and a key informant, explained that it is difficult to tell whether seeking Syrian brides by Egyptian men is, in fact, a prevalent phenomenon or if it is taken out of proportion. However, there were countless stories. She further stated that religious institutions such as Al‐Hossary Mosque, one of the largest mosques and charitable organizations in the Sixth of October City, were involved in matching Syrian women with Egyptian men under the justification of *sutra* or protection, which helped propel the issue to media and public attention. Some of the respondents referred to marriage as *sutra* (in another spelling *sotra*), an Arabic word meaning “to cover” that is often used to indicate protection or sheltering (see, for instance, Allassad Alhuzail, 2020; Taha, 2019; Acim, 2017). *Zawaj al‐sutra* (protection or shelter marriage) is a familiar notion, even if not practiced widely in Egypt. In such a case, the man is motivated to marry a widow, especially that of another man who died because of war, to provide her and her children with a livelihood and emotional support. Such practice is arguably recurrent throughout Islamic history, where many have suggested it was encouraged in the Islamic tradition. The community informants in this fieldwork agreed that some of the reasons Syrian women have always been viewed as desirable for marriage, not just by Egyptian men but also by men from the Arab world more broadly, are their compliance, femininity, and domestic skills as housewives. Their refugee status further exaggerated these traits.

## 4 DISPLACEMENT, GENDER NORMS, AND SHIFTING PERCEPTIONS TO MARRIAGE

Different discussions about identity (re)construction due to uprooting and displacement point to the importance of recognizing that the interaction between refugee experiences, location, and (re)interpretation of gender identity is often a strategic decision. Some studies have suggested a more complex expression of agency, where women demonstrate strategic or tactical agency (see Gale, 2007; Kim, 2014; Utas, 2005). For instance, Kim's (2014) study of women migration across the North Korea‐China border concluded that women “voluntarily and strategically use migration, marriage, and gender as arenas of agency through which to improve their lives and empower themselves” (p. 553). In such cases, women voluntarily, purposefully, and strategically use the structural barriers imposed on them because of their gender identity to acquire agency and control over their lives. Samuel (2013), on the other hand, discusses “strategic hybridity,” where she traces how Syrian Orthodox women migrants in Canada adapt and choose certain cultural aspects to display while hiding others. This begs the need to move beyond asking binary questions, such as whether a specific phenomenon is victimizing or an expression of agency. Instead, a more meaningful question is what enables some women in the same context to benefit from a phenomenon that has similar challenges and opportunities, while others cannot. An intersectional approach can assist in the understanding of this unevenness.

Refugee women adapt and reinterpret traditional gender roles and blur the line between the public and private spheres in response to displacement (Culcasi, 2019; Franz, 2003; Säävälä, 2010). Furthermore, migrant women often display greater resilience and adaptability by maintaining household and childcare routines during times of uncertainty (Szczepanikova, 2005). For Chechen asylum seekers living in a refugee camp in the Czech Republic, for instance, women's association with the private sphere is crucial to their coping strategies (Szczepanikova, 2005). In turn, joining the workforce did not necessarily liberate Bosnian refugee women who resettled in Vienna and New York where Franz (2003) demonstrated that their primary motivation was their families' survival and well‐being. Similarly, Al Sharmani's (2010) study on Somali refugees and migrants in Egypt from 2001 to 2004 highlights the dynamics within transnational family support systems. These systems involve reciprocal obligations, including the exchange of goods and shared care responsibilities, which play a critical role in the survival of these communities. In such arrangements, some Somali diaspora women assume the role of “the dependable daughter” as a survival strategy.

The transformation of gender roles, particularly with women's entry into the workforce, does more than just shift gender performances—it can also intensify traditional gender expectations. While employment may bolster women's influence within the home, it often adds to their burden by maintaining expectations of traditional roles, positioning their work as a means to juggle familial and maternal duties. Such dynamics suggest that certain liberal feminist views may not fully capture the complex realities of “family” and “motherhood”. For instance, El‐Masri et al. (2013) found that their Syrian and Palestinian refugee respondents' self‐worth was deeply tied to their traditional gender roles, noting a reluctance to disrupt these norms despite recognizing their limiting nature (p. 13). Culcasi (2019) echoed this sentiment, observing that displacement pushed Syrian refugee women into the workforce, challenging their traditional roles and femininity. The women in Culcasi's study perceived work as a distraction from their familial duties, fearing its impact on their children.

The interplay between marriage and displacement further complicates these dynamics. Research indicates that the meaning and structure of marriage evolve with cultural, contextual, and locational shifts. Gale (2007) explored the concept of Bulgur marriage among Sierra Leonean refugees in Guinean camps, highlighting how such transactional marriages accommodate multiple identities and enable social mobility, redefining conventional notions of marriage and kinship (p. 375). Similarly, Grabska (2010) examined how migration reshapes young South Sudanese refugee men who resettled in North America and their gender identities, dowry expectations, and attitudes toward marriage. As such, post‐displacement marriages are influenced by shifting gender and power dynamics, often used strategically by those displaced to optimize their standing in new social hierarchies.

Many studies discussing the topic of marriage and the Syrian refugee crisis fixate on GBV, early marriage, and forced marriage (Al Akash & Chalmiers, 2021; Bartels et al., 2018; El Arab & Sagbakken, 2019). Emerging research, however, is starting to recognize the centrality of marriage as a coping mechanism, agentive choice, and a response to displacement, moving beyond simplistic exploitation narratives (see, for instance, Lokot, 2024; Tobin, 2020; Taha, 2020; Akyuz & Tursun, 2019). For example, Achilli (2017) argued that portraying Syrian refugees as pure victims of exploitation by “violent men who prey on their vulnerability” is an oversimplified interpretation. Instead, it depicts a mixture of solidarity and mutual benefits (e.g., monetary benefits and enhanced refugee mobility) within a global legal system that promotes the securitization of borders, leaving refugees with little to no choice. Van Raemdonck (2021) offered a counterargument to the typical problematization of early marriage by local and international humanitarian agencies, suggesting that early marriage be considered a “desire for normality” and a continuation of a common practice in many regions in Syria before the war, which often see marriage as one's life course. Similarly, Zuntz et al. (2021) use the narratives of their female Syrian refugee interlocutors, including mothers and daughters, to paint a more complex picture than the humanitarian discourse. They explore how early marriage and marriage, in general, are perceived and negotiated by Syrian refugee women–mother and daughters, accounting for factors such as romance, compatibility, family obligations, and aspirations in marriage decision‐making. Zbeidy (2020) also highlights the centrality of marriage for Syrian refugees' post‐displacement futures, shaping marriage practices, meanings, and implications. Zbeidy emphasizes a preference among her interlocutors to marry fellow Syrians, “influenced by the desire to return to a post‐war Syria” (p. 73). Lokot's (2018) fieldwork with Syrian refugees in Jordan reflected similar sentiments of a preference to marry from one's governorate, emphasizing even more nuanced cultural and class distinctions among the Syrian population. This leaves a gap in understanding the motivations, experiences, and implications for those who marry from the host country.

## 5 RETHINKING THE MEANING, FORM AND PURPOSE OF MARRIAGE

Mohra, a 26‐year‐old widow with a child who married an Egyptian just a few weeks after arriving in Egypt, explained to me during our conversation how she weighed her options shortly after arriving in Egypt as a young widow and a single mother:

In my country, I had my rights and was able to manage. Here, I am in a strange country. Why would I work and degrade myself, meet this, and meet that, the good and the bad? No, I apply sutra [protection or sheltering] to myself and my daughter and find a human being who is honest and straightforward and offers me a decent life.

That is, displacement has (re)labeled marriage as a “decent” or even the only solution for some refugee women post‐displacement, especially after losing elements of their social capital such as cultural maneuverability, social connections, social status, and kinship (Bourdieu, 1989).

In the analysis below, I expand on the implications of displacement in redefining marriage while critiquing the prevailing colonial nuclear‐family‐centric assumption (Clark‐Kazak, 2011). I examine the different ways in which the meaning of marriage has undergone shifts for Syrian refugees, often driven by a mix of identity ruptures and strategic adjustments to adapt to the new reality of displacement and uprooting. The experiences of forced migration have reshaped marriage, its significance, and the circumstances under which it occurs. In response to displacement, some women gained an elevated sense of control, leading to the emergence of remarriage as a social option. Additionally, certain women have challenged the normative image of an ideal marriage, prompting the adoption of unconventional forms of marriage, namely, *‘urfi* (customary) marriage and polygamy. I address remarriage, *”urfi”* marriage, and polygamy respectively below.

### 5.1 (Re)marriage

Naziha, a mature Syrian refugee and mother of four who is married to an Egyptian man 5 years younger than her, sheds light on the transformative effects of displacement on her life choices and perceptions of gender relations. During our conversation, Naziha expressed how despite the hardships of uprooting, she found an unexpected degree of freedom from societal pressures and traditions. In Syria, her conservative middle‐class social context would have made remarriage an undesirable option for women such as her for several intertwined reasons:

Back in Syria, I wouldn’t have married for a second time. Why? Because I have a full community […] In Syria, you wish you have no commitments so that you can live without restrictions. We don’t have this idea about marrying for a second time. My ex‐in‐laws gave me a hard time. They started asking my children, ‘why did your mother marry? Your aunts did not get married again.’ Not Eib [shameful], just not common. After the first marriage, the woman’s community becomes her children and general socializing. You don’t need a second marriage. Life in Syria is beautiful, beautiful, beautiful.

The conservative community of Reef Demashk–a governorate that lies in the rural outskirts of Damascus where Naziha comes from, subjects middle‐aged women expressing interest in remarriage to scrutiny and disapproval since their existing social networks, encompassing their community, children, and extended family, had already fulfilled their support needs. Therefore, the idea of remarriage did not hold the same value as a source of social capital for women such as Naziha before displacement. Second, remarrying in Syria entailed socially accepted restrictions, including a husband's control over his wife's movements and decisions, which further dissuaded women from considering such a step. In her native social milieu, a woman who had been previously married but was currently single held a certain status of “*sitt mesh bint*”—a woman, not a girl—affording her more freedom compared to having a married status. Consequently, not only was remarriage socially frowned upon, but it also did not make practical sense given the level of freedom Naziha would have enjoyed in her single status in Syria. However, her displacement to Egypt brought about a shift in her perception of gendered relations, granting her greater power in her relationship with her current husband.

This newfound sense of power, control, and emancipation from socially ascribed gender relations became evident when she shared her strategy of asserting herself with her Egyptian husband, including techniques such as refusing to talk to him and not allowing him into the house until her demands, such as paying the rent for her apartment, were met. When asked if she would have employed the same methods as her previous husband in Syria, Naziha categorically stated that it would have been impossible. Her displacement changed her perception of emotional control and self‐reliance, empowering her to adopt previously incomprehensible methods to communicate with and influence her husband while maintaining her sense of control.

Naziha's experience was not unique among the respondents; many stated that they would likely not have considered remarriage had they stayed in Syria, especially after reaching a certain age and having children. In conservative areas such as Reef Damashk and in addition to the complex social dynamics described above, remarriage was also frowned upon, as it was perceived as betraying the sacred role of motherhood (see, for instance, Maman et al., 2019). Cultural and social factors also played a role in deterring some mothers from remarrying, for instance, the expectation that if a woman remarried, she would have to leave her children with the father's family. Others expressed concern about bringing a stepfather into their children's lives.

Another significant factor discouraging remarriage in Syria is the strong social support that these women usually enjoy from their families and friends. This support network provided them with social capital and a sense of safety and replaced the need for a spouse or partner, rendering remarriage unnecessary for many. Thus, for women such as Naziha, marriage represented a way to compensate for her loss of motherhood status rather than a practical consideration. Additionally, displacement disrupted the structural boundaries surrounding marriage and remarriage by removing some of the taboos surrounding the idea of a “mother” remarrying. That is, while the loss of social capital due to displacement did create vulnerabilities, some respondents strategically utilized their new circumstances to regain some of the lost support and work toward resettlement through remarriage, an option that would have had different considerations and restrictions before displacement.

### 5.2 *‘urfi* marriage

When the first wife of Marwa's husband asked her after the marriage secret was revealed, “Didn't you consider me? What would happen to me when my husband marries a second wife?” Marwa simply replied: “No, to be honest, I didn't consider you, I have enough on my plate. I did not have time to consider other aspects.”

One of the elements that causes significant criticism of seeking a Syrian bride in Egypt is that many of these marriages were reported to be *‘urfi* and polygamous. Many of the respondents I interviewed were married unofficially–limiting the marriage to the religious ceremony and hence not registered with the state through official paperwork or what is called *‘urfi* (customary) marriage. In Egypt, *‘urfi* marriage is generally used to circumvent traditional marriage obstacles and is often secretive and societally rejected. In Syria, customary marriage translates culturally into *shar'e* marriage or lawful/Islamically compliant marriage, as it often substitutes for the engagement step so that the bride and groom are allowed more time in private, and the bride can take off her headscarf if she wears one. It, thus, is surrounded by less stigma than the Egyptian context.

Many of the respondents involved in an *‘urfi* and a polygamous marriage arrangement challenged the narrative that perpetuates perceptions of this type of marriage as exploitative. By Egyptian standards, *‘urfi* marriage automatically implies an exploitative relationship often against the wife, since, for example, it is harder in unregistered marriages to prove marital and legal rights and even register children. However, many of the respondents defended “*urfi* marriage” by either equating it to legal or lawful marriage or justifying why *‘urfi* made the most sense in their situation. As Asmaa, a Syrian woman who became a second wife of an Egyptian man in secret using an “‘*urfi* contract”, explained, “You call it here [in urban Egypt] *‘urfi*, but it's like a regular paper [contract]. In Syria, this same paper can be registered in court.”

All of the interlocutors were aware of the difference between registered and unregistered marriages and that the ‘*urfi* marriage is less socially acceptable in Egypt than in Syria. They were also aware that it serves different purposes in the two countries: keeping the marriage secretive in Egypt versus serving as a substitute for engagement in Syria. Nevertheless, many women still found ways to justify the authenticity of the marriage and its morality and lawfulness. For instance, Asmaa justified:

I: So, your marriage was urfi

R: You call it here urfi, but it’s like a regular paper [contract]. In Syria, this same paper can be registered in court.

I: So, there were witnesses and an announcement of the marriage

R: Verbally only, like in the Prophet’s times with words. But you call it here urfi.

A central reason contributing to reshaping those women's perception of marriage is their need to maneuver many cultural and legal rules to secure a better social and economic position for their children and themselves. One of the main grounds for preferring a *‘urfi* marriage’ is that it makes a polygamous marriage easier and more discrete (i.e., secretive or unannounced). While polygamy is practiced to varying degrees in various Muslim societies and subcultures and social classes more than others, monogamous relationships and the nuclear family are still the most commonly accepted forms in most Egyptian urban communities and the majority of the Muslim Arab world^1^ (Al‐Krenawi & Graham, 2003). Many Egyptian wives resisted the idea of bringing in a second wife.

Thus, an *‘urfi* marriage offered some of my respondents economic and social benefits, at least within their social circle or neighborhood, while avoiding both the frequently stigmatized status of the second wife and the probable fighting and rivalry with the first wife. A fight that the new wife is not necessarily guaranteed to win. In such a case, keeping a “precarious” status, paradoxically, offers her more stability. Other women contested the meaning of secrecy to justify their moral grounding. Maha, for instance, a Syrian woman in her late forties who married an Egyptian man in an *‘urfi* arrangement, justified her and her husband's choice to opt for a *‘urfi* marriage that consequently had to be kept as a secret from the person who mattered the most: his first wife. In her assessment, secrecy helped protect his first wife from shock and humiliation while at the same time protecting the husband's social image and political career. When I tried to be more specific about the meaning of secretive marriage, Maha explained that technically it was not secretive since everyone knew except for his family: his wife and children and even the latter had their guesses.

Furthermore, contrary to their preferences if they were back in Syria, some Syrian women preferred to keep their marriages in Egypt *‘urfi*, making them unofficial and unregistered with the government, limited to a private religious ceremony. They justified this choice in various ways. First, some women feared that marrying an Egyptian would result in losing their legal refugee status with the UNHCR, making them ineligible for humanitarian financial assistance and food rations for themselves and their children. Second, by not registering the marriage with the Egyptian government, women retained more autonomy in case they later decided to end the marriage, leave the country, or return to Syria. The *‘urfi* or customary marriage simplified the separation process and allowed women to prove an unmarried marital status outside of Egypt at a later date. One respondent summarized:

I had no reason to register the marriage for any purpose, such as residency or money. I didn’t even want to register the marriage. I was worried he would turn out to be a bad guy, and I would have another bad experience because I trust no man anymore.

On the other hand, some women turned to *‘urfi* marriage as a way to navigate the legal system, which otherwise would not allow them to remarry. Particularly, some widows or divorcees faced challenges in proving that their previous marriages dissolved due to divorce or the death of their husbands in the war zone. The *‘urfi* marriage provided a solution for them, as it left no official legal trace and allowed them to move forward with their new relationships.

Nevertheless, during my conversations with many of the respondents, it was clear that they were wrestling with the complexity and contradictions inherent in choosing unconventional forms of marriage such as *‘urfi* marriage. Despite the challenges, these women view their *‘urfi* unions as complete marriages and find moral alignment in their decisions. For instance, Naziha's previous negative experience and as we will see next Latifa's moral dictations and Luli's pragmatic calculations shaped their choice of entering an *‘urfi* polygamous marriage, where they emphasized the importance of “fair treatment” for her husband's first wife and children as a condition for the marriage.

### 5.3 Polygamy

Although most, if not all, of the *‘urfi* cases in my sample were also polygamous, the latter offered different benefits and required different reasoning in the marriage arrangement. Luli is a young mother of two who comes from a well‐off family and is highly educated with a very active social life. She explained to me how she often felt suffocated by her current Egyptian husband's controlling tendencies even though she loves and respects him. Luli used to work before she met her husband and had been in a previous failed marriage where she and her ex‐husband had very independent lifestyles. When her current husband met her, he was already married and had a family. Luli encouraged him to stay with his family and to continue to treat them well. She believed it was in her best interest to be a “part‐time wife,” where her husband is responsible for two households and is only available to her part of the week. She noted that after her husband split from his first wife recently, she lost much of her freedom as he became a full‐time resident of her house and directed all of his energy and attention toward her:

I thought the problem [of his controlling tendencies] would be divided [between the two households] … I swear to Allah I begged him not to leave them [his first family] and to make amends with his wife. He would say, ‘This is a better situation for me; she turned my life to hell.’ Sometimes they make amends, sometimes they split. For the past month, the situation has become so bad, so I felt that the whole focus has turned to me.

Luli was explicit that the main reason behind encouraging her husband to keep his first house intact was for her to have more freedom and more time with her children, one of whom is from her current marriage. For Luli, polygamy served her own personal interest.

On the other hand, Latifa reiterated the advantage of “part‐time” marriage and was convinced that polygamy is a healthy form of marriage and is thus encouraged religiously. She even explained that her husband's first wife was the one who set them up:

I didn’t agree until I knew the opinion of his wife. He swore to me that she was the one who was seeking a wife for him. I told him I need proof; I need to meet her face to face; otherwise, it won’t work out. Therefore, So, he agreed to let us both meet and then Subhan Allah [an expression to reflect exclamation] now we are both friends, even more than him.

Latifa's rationale reflects the different factors influencing her subject formation and, ultimately, her agentive choice to agree to become a second wife. It shows how her ethical framework represented in her need to seek the first wife's approval is shaped by moral and ethical convictions. Latifa elaborates on this “Othered” meaning of marriage and the benefits of polygamy from a religious perspective, as she explains it to be “the natural course of life…That the man would have two, three or four wives at the same time.” That said, as Latifa elaborates on how she feels, we notice that she understands that she benefits from the marriage, and we see her moral and ethical reasoning eventually come together with her personal interests. That is, self‐interest as a pillar of exercising agency was still among one of the factors that shaped her final decision:

Listen, if he [the husband] is fair, then it is his right because this is a Sunnah^2^ from the Prophet. In addition, in fact, this arrangement removes a lot of burden from your shoulders. When he is at my house day in and day out, he will get used to me and know all my secrets. However, when he is not around every day, you become more comfortable around the house. When he comes back again, he will find you different [referring to boredom in marital life] … because the man is more visual than verbal, so he might get used to you.

Luli and Latifa come from completely different socioeconomic and demographic backgrounds. Luli is in her late twenties, does not wear a *hijab*, and demonstrates opinions and a lifestyle that reflect fewer conservative beliefs than Latifa. Luli is also very well‐educated and comes from a well‐off family. On the other hand, Latifa, who is well accomplished careerwise, showed a deep commitment to socially ascribed Islamic values and compliance with conservative interpretations of Islamic *sharia*. She is in her fifties and from a well‐educated yet lower‐middle‐class environment. Nevertheless, both women agreed on the same “part‐time husband” logic as one of the benefits of polygamy, that is, that it allows them more freedom and reduces their marital responsibilities. However, Latifa assigned polygamy a moral and religious grounding by emphasizing how it maintains a healthy relationship between spouses, reducing boredom from seeing each other every day. It also reduces, in her view, the chances of friction and confrontation. This practical point of view is her way of supporting her understanding of moral and religious obligations. As she rationalized, Islamic law would never allow any harmful practices, so it must have allowed polygamy for the husband for a good reason to reflect a healthy family and a healthy society. Nevertheless, both women had their points of view reinforced by their realities. While Latifa is leading a successful life with her “sister wife” and husband as they try to maintain “their partnership,” Luli started to suffer after her husband left his first wife. Though they both agree on the logic, a pragmatic versus a religious rationale makes a difference in marriage and gender dynamics, especially given Latifa's assumptions regarding the different natures of men and women.

## 6 DISCUSSION

While displacement has exacerbated the patriarchal conditions that refugee women must navigate, it has also prompted some Syrian women to reevaluate the purpose of marriage. This has made (re)marriage a viable option once again for certain stigmatized groups (e.g., mothers) and employing it strategically for self‐resettlement and to reclaim their lost social capital. For instance, in Egypt, cultural and legal constraints have influenced some women's preference for *‘urfi* marriage due to its discreet nature, especially in polygamous situations. Despite its precarious nature, this choice offers stability by simplifying separation and thus supporting women's autonomy in ending the marriage whenever they wish, exemplifying a strategic choice and a calculated risk.

Nevertheless, the stories of the interlocutors reveal the complex web that shapes their choices, which is driven not just by self‐interest but also by ethical and moral frameworks as well as an embracing of traditional gender roles. I thus contend that limiting our analysis to a liberal understanding of agency, often understood as resistance to different forms of domination, the ability to subvert norms and the capacity to realize one's own interest against custom (see, for instance, Asad, 1996; Butler, 1990; Bilge, 2010), can severely restrict our comprehension of the complete experiences and subjectivities of some refugee women. Instead, I argue that these women's decisions and desires to marry are determined by a complex web shaped by: (a) a liberal conception of agency, weighing personal interests against customs; (b) relational autonomy while navigating patriarchal expectations that redefine marriage as the decent and almost the sole solution; and (c) the women's moral agency. This moral agency does not primarily aim to enhance material interests or status but rather to “attain a certain kind of state of happiness, purity, wisdom, perfection, or immortality” (Mahmood, 2006, p. 210).

We can see a striking example in Latifa when she rationalized her decision to become a second wife, basing it on factors such as (a) self‐interest, as it frees her from her husband's constant scrutiny; (b) relational autonomy, reflecting her choices within a patriarchal society shaped by mutual dependencies, and compensating for the loss of social capital, particularly the motherhood status that many women rely on to navigate patriarchy and gain status; and (c) her moral agency, represented in religion and her ethical interpretation of religious texts and principles as well as her valuing of certain aspects of traditional gender roles.

Similarly, Marwa's response to her husband's first wife reveals a lack of consideration for the first wife's feelings when calculating the cost and benefit of the marriage. However, a significant fracture in this “pursuit of self‐interest” narrative is that these women still actively identify with the traditional marriage institution and many patriarchal discourses. This fracture is echoed by some respondents, such as Maha's statement that “a woman without a man is like a tree without leaves,” Nour's belief that a woman's ultimate path is marriage, and Marwa herself and her choice of marriage over monthly financial support when given the option by her current husband.

Butler (1990) argued the modes that allow agency are, in fact, the products of power operations. She locates the possibility of resistance to norms, and any act of agency for that matter, within the structure of power itself “rather than in the consciousness of an autonomous individual” (Mahmood, 2001, p. 211). A decolonial reading, thus, can help us emphasize the social construction aspect of agency, particularly the role of norms and structures in (re)producing agency and subjectivity, as well as the analytical value of separating agency from resistance to understand certain Othered experiences. The analysis reveals that in many cases, the respondents perceive marriage not merely as a means for socioeconomic advancement or navigating social structures, but as an ethical and virtuous choice that resonates with their personal beliefs and gender identities.

That said, while I argue in this research that marriage should be considered a practical, “decent,” and culturally relevant solution for many refugee women who are “bargaining with patriarchy” (Kandiyoti, 1988) including single mothers, it cannot be viewed in isolation from other cultural norms and discursive powers that have shaped these women's consciousness. This paper is thus not oblivious to the patriarchal and unjust conditions, such as fear of harassment or concerns about personal safety, that underlie the socio‐cultural milieu of these women and influence their preferences and decisions to marry. Instead, as Mahmood (2001) eloquently puts it: “In order for us to be able to judge, in a morally and politically informed way, even those practices we consider objectionable, it is important to take into consideration the desires, motivations, commitments, and aspirations of the people to whom these practices are important” (p. 225).

Insecurity and precarity create circumstances that give rise to specific types of relationships, challenging Western‐Eurocentric family ideals and the emotional expressions typically associated with them such as exclusivity (Balakian, 2023). The upheaval and loss of social capital after displacement lead many women to view marriage as a means to regain stability, social status, and long‐term security. A decolonial lens allows us to move beyond reductive narratives of exploitation, revealing how certain marital bonds defy dominant views on the conventional nuclear family, intimacy, and love as marriage's sole motivators. The analysis underscores the importance of a decolonial approach in challenging entrenched notions of the ideal family and marriage, and in recognizing agentive choice traditionally viewed through a liberal lens—primarily as resistance to norms—as potentially embodying both strategic and genuine acceptance of traditional roles.

## 7 CONCLUSION

Displacement almost always depletes social capital, leaving some respondents vulnerable but also presented different considerations and alleviated some social restrictions compared to before displacement. Marrying again after becoming a widow or a divorcee became a viable option for social and pragmatic reasons while simultaneously coupled with moral reasons. *‘urfi* marriage and polygamy, despite being socially less accepted, have presented women with alternative paths to navigate displacement, gender dynamics in the new context and exercise agency in their lives. The evolving understanding of marriage in the context of displacement reflects a complex interplay between cultural, religious, and individual factors, ultimately shaping the dynamics of gender relations in these communities. The narratives of the women in this study challenge conventional humanitarian and liberal perspectives on marriage, revealing the malleable nature of marital and family structures beyond Western norms. While pragmatic considerations such as flexibility and financial benefits play a role in their decisions, their understanding of marriage and gender identity goes beyond strategic maneuvering; rather, it emerges from their culturally ascribed gender roles, posing clear challenges to the liberal connotation of agency with subversion of norms.

Furthermore, by examining the complex layers of agency within the context of marriage in displacement, it is important to recognize how deeply embedded social norms and structures shape personal choices and identities. It highlights that for these women, marriage transcends socioeconomic motives, embodying an alignment with ethical values and traditional gender identities. This perspective challenges us to reconsider the dynamics of power and agency, suggesting that what might appear as conformity may in fact represent a complex navigation of societal expectations and personal convictions.

This study expands discussions on gender, displacement, and marriage, challenging prevailing norms and offering new perspectives beyond colonial binary categories (e.g., agent/victim, Exploited/empowered, marriage for love/marriage for interest). The intertwining of marriage and resettlement shows how marriage becomes a self‐initiated form of resettlement, and *re‐rooting*, providing legal, economic, and crucial social and moral support. The prevailing notion of a “real” marriage is thus decentered, emphasizing the significance of extended family dynamics and leveraging socially ascribed gender roles in seeing marriage as a way to restore stability, status, and security. Nevertheless, intimacy and creating a family were not mutually exclusive from other pragmatic considerations, including resettlement and rebuilding their social capital, and were not in a hierarchical relationship. Rather for some, marriage was not meant to create new families but to preserve existing ones. That is, the meaning of marriage, along with if, how, and when it takes place, has been repurposed to serve those women's new circumstances and status as uprooted, unraveling an Othered perspective on conjugal relations.

## CONFLICT OF INTEREST STATEMENT

The authors report that there are no competing interests to declare.

## ETHICS STATEMENT

This research has received ethics review and approval by the Human Participants Review Sub‐Committee, York University's Ethics Review Board and conforms to the standards of the Canadian Tri‐Council Research Ethics guidelines.

## Data Availability

The data that support the findings of this study are available from the corresponding author upon reasonable request.

## ENDNOTES

^1^
Al‐Krenawi and Graham (2003) estimate that polygamous households represent only 2–11.5 percent of all in the Muslim Arab world.

^2^
In Islam, Sunnah refers to the traditions, actions, sayings, and approvals of the Prophet Muhammad (peace be upon him). It serves as a significant source of guidance for Muslims, complementing the teachings of the Quran. The Sunnah provides insights into the practical application of Islamic principles and serves as a model for Muslims to follow in their daily lives, fostering a deeper understanding of faith and promoting righteous conduct.
